# FISH mapping in *Xenopus pygmaeus* refines understanding of genomic rearrangements and reveals jumping NORs in African clawed frogs

**DOI:** 10.1038/s41437-025-00749-x

**Published:** 2025-03-01

**Authors:** Barbora Bergelová, Václav Gvoždík, Martin Knytl

**Affiliations:** 1 Department of Cell Biology, Charles University, Viničná 7, Prague, 12843 Czech Republic; 2 Institute of Vertebrate Biology of the Czech Academy of Sciences, Brno, Czech Republic; 3 Department of Zoology, National Museum of the Czech Republic, Prague, Czech Republic; 4 Department of Biology, McMaster University, 1280 Main Street West, Hamilton, L8S4K1 ON Canada

**Keywords:** Molecular evolution, Cytogenetics, Polyploidy

## Abstract

Chromosomal rearrangements are fundamental evolutionary drivers leading to genomic diversification. African clawed frogs (genus *Xenopus*, subgenera *Silurana* and *Xenopus*) represent an allopolyploid model system with conserved chromosome numbers in species with the same ploidy within each subgenus. Two significant interchromosomal rearrangements have been identified: a translocation between chromosomes 9 and 2, found in subgenus *Silurana*, and a fusion between chromosomes 9 and 10, probably widespread in subgenus *Xenopus*. Here, we study the allotetraploid *Xenopus pygmaeus* (subgenus *Xenopus*) based on in-depth karyotype analysis using chromosome measurements and fluorescent in situ hybridization (FISH). We designed FISH probes for genes associated with translocation and fusion to test for the presence of the two main types of rearrangements. We also examined the locations of 5S and 28S ribosomal tandem repeats, with the former often associated with telomeric regions and the latter with nucleolus organizer regions (NORs). The translocation-associated gene mapping did not detect the translocation in *X. pygmaeus*, supporting the hypothesis that the translocation is restricted to *Silurana*, but instead identified a pericentromeric inversion on chromosome 2S. The fusion-associated gene mapping confirmed the fusion of chromosomes 9 and 10, supporting this fusion as an ancestral state in subgenus *Xenopus*. As expected, the 5S repeats were found predominantly in telomere regions on almost all chromosomes. The nucleolar 28S repeats were localized on chromosome 6S, a position previously found only in the closely related species *X. parafraseri*, whereas other, phylogenetically more distant species have NORs located on different chromosomes. We therefore hypothesize that a jumping mechanism could explain the relatively frequent changes in the location of NORs during *Xenopus* evolution.

## Introduction

Amphibians are a group of small vertebrates with highly diverse ploidy levels, ranging from diploid to dodecaploid (Mezzasalma et al. 2023), but at the same time their genomes are highly conserved in terms of karyotype structure, synteny between orthologous chromosomes, and low rates of chromosome rearrangements (Bredeson et al. 2024; da Silva et al. 2021; Teixeira et al. 2016; Uno et al. 2013). Due to the strong stability of chromosomes, genome evolution in frogs has been described as “slow” (Bredeson et al. 2024). The exceptions that represent karyotypic structural innovations are Robertsonian translocations and end-to-end fusions (Bredeson et al. 2024), considered to be a driving force of frog evolution in general (Morescalchi 1973).

The frogs of the family Pipidae include four genera, *Xenopus*, *Hymenochirus*, *Pseudhymenochirus*, and *Pipa*. Among these, at least two genera (*Xenopus* and *Hymenochirus*) consist of both diploid and polyploid species (Gvoždík et al. 2024; Tymowska 1991). In addition, pipids involve two model species – the diploid *Xenopus tropicalis* and the tetraploid *X. laevis* – frequently used for biological and biomedical research (Cannatella and de Sá 1993; Tandon et al. 2017), whose high-quality chromosome-scale genome assemblies have been generated in several updated versions (Bredeson et al. 2024; Hellsten et al. 2010; Mitros et al. 2019; Session et al. 2016). Furthermore, pipid frogs include other emerging models, *X. borealis*, *Hymenochirus* sp. and *Pipa parva* with draft genome sequences available (Bredeson et al. 2024; Cauret et al. 2020; Evans et al. 2022).

Genus *Xenopus* contains two subgenera, *Xenopus* and *Silurana* (*sensu* Evans et al. 2015) with a deep evolutionary divergence 45–50 million years ago (Mya) (Feng et al. 2017; Session et al. 2016). The only described diploid species within entire genus, *X. tropicalis*, belongs to subgenus *Silurana*, along with three tetraploids: *X. calcaratus*, *X. mellotropicalis*, and *X. epitropicalis*. *Xenopus tropicalis* has 20 chromosomes, which is considered the ancestral chromosome number in the whole pipid family (Bredeson et al. 2024; Mezzasalma et al. 2015). Tetraploids in subgenus *Silurana* have 40 chromosomes and their genomes arose from a fusion of two diploid ancestors with 20 chromosomes (Chain et al. 2008; Evans et al. 2004). The complements of the ancestral genomes, referred to as subgenomes, can be distinguished from each other based on sequencing and cytogenetic methods (Evans et al. 2005; Knytl et al. 2017, 2023; Session et al. 2016). *Silurana* subgenomes are referred to as a- and b-subgenomes (*sensu* Knytl et al. 2017) and sometimes as alpha- and beta-subgenomes (*sensu* Evans et al. 2005). The a-subgenome is more closely related to the *X. tropicalis* genome than the b-subgenome (Evans et al. 2005, 2015). Subgenus *Xenopus* is more diverse than subgenus *Silurana* in terms of species diversity and ploidy levels. This subgenus comprises 25 species and three ploidy levels (tetraploid, octoploid, dodecaploid) (Evans et al. 2015; Tymowska 1991). The subgenomic units in subgenus *Xenopus* are called L- and S-subgenomes (Longer, Shorter; *sensu* Matsuda et al. 2015). The L-subgenome is more stable and thus more resistant to reorganization, and therefore there is a less divergence between the L-subgenome and the *X. tropicalis* genome than between the S-subgenome and the *X. tropicalis* genome (Lau et al. 2020). The S-subgenome is more likely to undergo chromosomal rearrangements and gene loss than the L-subgenome (Session et al. 2016). All tetraploid species within subgenus *Xenopus* have 36 chromosomes, octoploids have 72 chromosomes, and dodecaploids have 108 chromosomes (Evans et al. 2015; Tymowska 1991). All polyploids in genus *Xenopus*, including those in subgenus *Silurana*, are allopolyploids, whose genomes were derived from distinct parental species (Evans et al. 2005, 2015; Knytl et al. 2023; Session et al. 2016; Tymowska 1991). The divergence time of the a- and b-subgenomes (parental species) in *Silurana* is estimated to be 10 Mya (Evans et al. 2015), while in subgenus *Xenopus* it is estimated to be 30–35 Mya (Session et al. 2016). The allotetraploidization event in the latter subgenus occurred at least 17–18 Mya, with subsequent polyploid radiation starting at least 17 Mya and forming three major clades, with the *X. muelleri* species group branching first, and the *X. laevis* and *X. amieti* species groups split around 10–17 Mya (Evans et al. 2015, 2024; Fornaini et al. 2023; Session et al. 2016).

Despite the relatively conserved karyotypes of pipid frogs, two interchromosomal rearrangements have been identified—one in each subgenus. In *Silurana* (*X. mellotropicalis*), a non-reciprocal translocation of the pericentromeric region between chromosome 9b and 2b has been discovered (Knytl et al. 2017, 2018). The a- and b-chromosomes are partitions of the a- and b-subgenomes, respectively, as well as the L- and S-chromosomes within the L- and S-subgenomes (Knytl et al. 2017; Matsuda et al. 2015). Accordingly, the translocation occurred in the b-subgenome of *X. mellotropicalis* but did not occur in diploid *X. tropicalis* (Knytl et al. 2017, 2018). It has not been tested whether this translocation is shared with species of subgenus *Xenopus*. In subgenus *Xenopus*, another interchromosomal rearrangement has been described between ancestral *X. laevis* chromosomes 9 and 10, which fused to form chromosomes 9_10L and 9_10S (Session et al. 2016). The fusion likely occurred in the diploid state of an ancestor of subgenus *Xenopus* (Session et al. 2016) and is therefore assumed to be present in all other species of subgenus *Xenopus*. However, the fusion has not been thoroughly investigated to detect breakpoints and fusion junctions in species of subgenus *Xenopus* other than *X. laevis*.

Genome reorganization can also occur through clusters of repetitive DNA deposited as tandem repeat units, sometimes referred to as multigene families (Dias et al. 2024; Sember et al. 2020). These units undergo concerted evolution, a process that leads to homogenization and uniformity of paralogous copies across the genome (Wang et al. 2023). One example of tandem repeats is ribosomal DNA (rDNA), which may (nucleolar, e.g., 28S rDNA) or may not (non-nucleolar, e.g., 5S rDNA) be associated with the formation of a nucleolus, specifically, a nucleolus organizer region (NOR) (Fornaini et al. 2024; Symonová and Howell 2018). Traditionally, NORs have been localized in genus *Xenopus* using conventional cytogenetic techniques like C-banding or silver nitrate staining (Tymowska 1991), but these techniques often target heterochromatic blocks and may not be efficient enough to detect active NORs that are sometimes euchromatic or inactive in the previous interphase of the cell cycle (Dobigny et al. 2002; Sánchez et al. 1995; Unal Karakus et al. 2024). A plausible solution for detecting true NORs is fluorescent in situ hybridization (FISH).

In this study, we examine the allotetraploid Pygmy clawed frog, *X. pygmaeus* (subgenus *Xenopus*, *X. amieti* species group, 2n = 36), with the aim to better understand genome evolution by chromosomal rearrangements in genus *Xenopus*. We question (1) whether *X. pygmaeus* has the same 9-2 interchromosomal translocation as *X. mellotropicalis* (subgenus *Silurana*), and/or (2) the 9-10 fusion as found in *X. laevis* (subgenus *Xenopus*), and/or (3) whether there is another rearrangement that may drive genome evolution in *X. pygmaeus*. In order to answer the above questions, we use FISH mapping of ribosomal (linked to NORs) and selected single-copy genes to provide a detailed resolution of the *X. pygmaeus* karyotype. We then compare our mapped locations with the locations of the same genes in the allotetraploid *X. laevis* from the same subgenus (*X. laevis* species group, divergence about 10–17 Mya), and the diploid *X. tropicalis* and allotetraploid *X. mellotropicalis* from subgenus *Silurana* (divergence 45–50 Mya).

## Materials and methods

### Origin of samples and preparation of primary cell cultures

*Xenopus pygmaeus* from the Democratic Republic of the Congo (Kokolopori, Yalokole, near Luo River, 0.2056^∘^N, 22.8884^∘^E) was used in this study and is the only representative of genus *Xenopus* that has been found in this region (Badjedjea et al. 2022). A parental couple was reproduced without any hormonal stimulation. IDs of mother and father are CD18.538 and CD18.537b, respectively (Evans et al. 2024). Primary cell cultures were established from the hindlimbs of four tadpoles at stage NF55 (± 1) (Sinzelle et al. 2012), for *Xenopus* stages see https://www.xenbase.org/xenbase/anatomy/alldev.do. The tadpoles were anesthetized using MS-222. IDs of the four tadpoles used for cell cultures are XPYTaF1-1–XPYTaF1-4. The composition of the cultivation medium was the same as described in Knytl et al. (2023). The cells were cultivated at 29.5^∘^C with 5.5% CO_2_. The medium was changed daily for one week, and then three times a week thereafter (Fornaini et al. 2023). Passages were performed with trypsin-ethylenediaminetetraacetic acid (Knytl et al. 2017). For cell cultures, we used siblings from the same cross. Other siblings of the same cross were grown and used as probes for single-copy gene mapping (ID XPYTaF1-5). The parents of the studied tadpoles were genotyped by reduced representation genotyping by sequencing and sequencing a portion of the 16S ribosomal RNA (rRNA) gene in the mitochondrial genome (Evans et al. 2024), which confirmed the identity of the species.

### Analysis of karyotype

Chromosomal suspensions were prepared from cell cultures according to the protocol described by Khokha et al. (2009) with minor changes (Krylov et al. 2010). Ready-to-use suspensions were stored in a methanol:acetic acid 3:1 fixative solution at –20^°^C. Chromosomal suspension was dropped onto microscope slides according to Courtet et al. (2001) and FISH procedures followed. Each sample was stained using Pro-Long® Diamond Antifade Mountant with the fluorescent 4’,6-diamidino-2-phenylindole, DAPI stain (Invitrogen by Thermo Fisher Scientific, Waltham, MA, USA) to label each chromosome in a metaphase. For analysis of karyotype, short (p) and long (q) arms of each chromatid were measured in pixels using ImageJ, V 1.53k (Schneider et al. 2012). The lengths of the p and q arms were quantified as described in Knytl and Fornaini (2021). To identify each chromosome, we calculated chromosome length (*l*), centromeric index (*i*), and p/q and q/p arm ratios (*r*_1_, *r*_2_) (Levan et al. 1964; Tymowska 1991); see Knytl et al. (2023) for formulas. Each measured chromosome was compared to its corresponding chromosome in the *X. pygmaeus* karyotype assigned by Tymowska (1991). However, the chromosome arms were not measured by Tymowska (1991), and therefore, we used measurements from *X. laevis* as a template (Matsuda et al. 2015; Tymowska and Kobel 1972) for chromosome identification in *X. pygmaeus*. Chromosome nomenclature was taken from Matsuda et al. (2015). Each individual chromosome was also assigned a chromosomal category based on the *i* value. If the *i* value was equal to or greater than 37.5, the chromosome was categorized as metacentric. If the *i* value was equal to or higher than 25 and lower than 37.5, the chromosome was categorized as submetacentric, and if the *i* value was equal to or greater than 12.5 and lower than 25, the chromosome was categorized as subtelocentric. Data were analyzed in R software for statistical computing, V 4.3.1 (R Core Team 2020) using the ggplot2 and ggpubr packages. To test whether the *l* and *i* values differed significantly between two homeologous chromosomes, one-way analysis of variance (one-way ANOVA) and Tukey’s test were performed using R scripts modified from Knytl and Fornaini (2021). All steps outlining how the measured values were calculated and processed into tables and plots, including metaphase images, are available on https://github.com/martinknytl/2024_pygmaeus_karyotype. Eleven metaphase images were analysed for karyotype analysis; ten to 20 metaphase images were analysed in each FISH experiment.

### Mapping of 28S and 5S ribosomal genes

Laboratory strain of *X. tropicalis* (Ivory Coast), long-term bred at Charles University in the Czech Republic, was used as a source for amplification of 28S and 5S rDNA probes. Total genomic DNA was extracted from the liver of adults using the DNeasy Blood & Tissue Kit (Qiagen, Hilden, Germany) according to the manufacturer’s instructions. Primer sequences for 28S and 5S (Integrated DNA Technologies, Coralville, IA, USA) are listed in Table 1. Identical *X. tropicalis* 5S (100% identity with accession number OR360596) and 28S (100% identity with accession numbers XR_004223792–XR_004223798) sequences from Gvoždík et al. (2024) and Knytl and Fornaini (2021), respectively, were used and labeled. The preparation of the 28S and 5S probes including PCR conditions, labeling with digoxigenin-11-dUTP and biotin-16-dUTP (both Jena Bioscience), respectively, and purification, is detailed in Knytl & Fornaini (2021). The 28S and 5S probes were hybridized with chromosomal spreads of *X. pygmaeus*. The composition of the hybridization mixture and the hybridization process were conducted as detailed in Knytl et al. (2023). Post-hybridization washing and blocking reaction were performed as described for painting FISH in Krylov et al. (2010). The probe signal was visualized as described in Knytl et al. (2017).

**Table 1:** Species names, markers used in FISH analyses, organs used for DNA and RNA extractions, PCR primer sequences, lengths of amplicons, working designation of plasmid clones selected for FISH, GenBank accession numbers, and references to studies in which the primers were designed.

Species	Gene symbol	Name of the gene	Tissue	Primer sequence	Size (bp)	Clone	Accession number	Reference
***Xenopus tropicalis***	28S	28S ribosomal RNA	liver	28SA: 5’-AAACTCTGGTGGAGGTCCGT-3’	250	–	–	Naito et al. (1992)
				28SB: 5’-CTTACCA AAAGTGGCCCACTA-3’				
	5S	5S ribosomal RNA		XTR 5S F: 5’-AATGGGAGCGCACCTACCT-3’	251	–	–	Knytl and Fornaini (2021)
				XTR 5S R: 5’-CATCCACAATGCACTGGTCT-3’				
***Xenopus pygmaeus***	*fn1*	fibronectin		Xen_fn1_F1: 5’-AATGGGAGCGCACCTACCT-3’	1161	B	PQ438168	this study
				Xen_fn1_R1: 5’-CATCCACAATGCACTGGTCT-3’				
	*sf3b1*	splicing factor 3b subunit 1	lung	Xen_fs3b1_F: 5’-TTGCGAAAACACACGAAGATATTG-3’	1458	A	PQ438169	
				Xen_fs3b1_R1: 5’-GTGTAGATTCATCAACATCAACCA-3’				
	*ndufs1*	NADH:ubiquinone oxidoreductase core subunit S1	liver	Xen_ndufs1_F: 5’-AGAAACCCATTGTGGTGGTTG-3’	982	A	PQ438170	
				Xen_ndufs1_R2: 5’-GGTAATAAACCATTTTAATAAGTTTACAAG-3’				
	*cept1*	choline/ethanolamine phosphotransferase 1	lung	Xen_cept1_F: 5’-GTGGAAATCCCTACCAAACAG-3’	1190	E	PQ438171	
				Xen_cept1_R2: 5’-CACTTAGTGATGATTAAAACGTGC-3’				
	*gyg2*	glycogenin 2		Xen_GYG2_F: 5’-TACTGTCAAGGAGCCCTGG-3’	1298	C	PQ438172	
				Xen_GYG2_R2: 5’-GCAAAACTAAATAGCTCTAATTTATTTG-3’				
	*bmp7*	bone morphogenetic protein 7	spleen	Bmp7_F: 5’-GAGAAGGGCAGGTGCTGTAG-3’	1211	B	PQ438173	
				Bmp7_R: 5’-TGGCTTTGGAACAGTGTCTG-3’				
	*nomo3*	NODAL modulator 3		NOMO3_F: 5’-GAGTTTCGCTTTGAGCCATC-3’	1165	C	PQ438174	
				NOMO3_R: 5’-AAGGTCCAGGGTCCTCAAGT-3’				
	*sox9*	SRY-box transcription factor 9	liver	SOX9_F: 5’-GAGAACACCAGACCCCAAGA-3’	985	B	PQ438175	
				SOX9_R: 5’-CTGTTGCTGCTGGTCACTGT-3’				

A dash in the clone column provides information that the amplicons were not cloned.

A dash in the accession number columnindicates that the sequences were not uploaded to the GenBank database.

### Mapping of single-copy genes

RNA was extracted from multiple organs from adult *X. pygmaeus* offspring (Table 1) using the E.Z.N.A. Total RNA Kit I (Omega Bio-tek; Norcross, GA, USA). RNA was reverse-transcribed into cDNA using the RevertAid H Minus First Strand cDNA Synthesis Kit (Gibco by Thermo Fisher Scientific). Eight autosomal genes were amplified: choline/ethanolamine phosphotransferase1 (*cept1*), glycogenin 2 (*gyg2*), fibronectin 1 (*fn1*), NADH: ubiquinone oxidoreductase core subunit S1 (*ndufs1*), splicing factor 3b subunit 1 (*sf3b1*), NODAL modulator 3 (*nomo3*), bone morphogenetic protein 7 (*bmp7*) and SRY-box transcription factor 9 (*sox9*). Primers were designed based on available orthologous sequences of *X. laevis* and *X. tropicalis*. All primers, along with their corresponding gene names, are summarized in Table 1. Justification for gene selection is given in Section Results, Mapping of single-copy genes. Amplified genes were cloned using the TOPO-TA cloning kit (Invitrogen, Camarillo, CA, USA). Plasmid DNA was isolated from 4–5 bacterial colonies for each gene using the E.Z.N.A. Plasmid DNA Mini Kit I (Omega Bio-tek), checked by electrophoresis, and sent for sequencing. Sequences were compared with sequences from *X. laevis* using Geneious Prime, version 2023.0.4. The cDNA sequences were deposited in the GenBank database (Table 1).

The FISH with tyramide signal amplification (FISH-TSA) protocol was adopted from Krylov et al. (2007) with minor modifications as described in Knytl et al. (2018). FISH probes were labeled with digoxigenin-11-dUTP (Roche, Mannheim, Germany), signal was detected by antidigoxigenin-POD, Fab fragments (Roche), and amplified using the TSA TM-Plus Tetramethylrhodamine System Kit (NEL742001KT, PerkinElmer, Inc., Waltham, MA, USA).

### Microscopy and processing of FISH images

Fluorescence FISH images were captured using a Leica DM6 upright microscope coupled with fluorescence units, a Leica DFC7000 T camera, and Leica Application Suite X software, version 3.7.3.23245 (Leica Microsystem, Wetzlar, Germany). Images from double-color FISH with ribosomal probes were acquired in three different channels: blue (DAPI), red (28S probe with biotin and streptavidin-Cy3), and green (5S probe with digoxigenin and anti-digoxigenin-fluorescein). Images from single-color FISH-TSA were captured in two channels: blue (DAPI) and red (single-copy gene probes with digoxigenin and anti-digoxigenin-peroxidase-rhodamine). The red-green-blue (RGB) and red-blue channels were merged using Adobe Photoshop, version CS7 (San Jose, CA, USA).

## Results

### Analysis of karyotype

All four individuals consistently had 36 chromosomes (2*n* = 4*x* = 36), where *n* denotes the haploid chromosome number of the extant species, and *x* denotes the haploid chromosome number of the most recent diploid ancestor of the extant species. This finding confirms the biological diploidy and evolutionary paleotetraploidy of all individuals analysed, which coincides with Tymowska (1991).

The arms of each chromosome were measured from 11 metaphase spreads and the median values of *l*, *r*_1_ and *i* were calculated. The value of *l* was quantified as a percentage of the sum of *l* for all chromosomes to account for variation in resolution and pixel size of our images. The karyotype of *X. pygmaeus* consists of 10 pairs of metacentric (m), 1 pair of submetacentric (sm), and 7 pairs of subtelocentric (st) chromosomes (Table 2). Neither acrocentric chromosomes (*i* interval 0–12.5) nor telocentric chromosomes (no p arm; *i* = 0) were present in the *X. pygmaeus* karyotype.

**Table 2:** *Xenopus pygmaeus* chromosome characteristics for each haploid chromosome shown in the medians of chromosome length (*l*), p/q arm ratio (*r*_1_), and centromeric index (*i*).

Chromosome	l (%)	r_1_	i	Category
1L	4.25	0.70	41.35	m
1S	3.69	0.68	40.49	m
2L	3.45	0.61	37.82	m
2S	3.09	0.56	35.85	sm
3L	2.94	0.25	20.00	st
3S	2.74	0.28	21.97	st
4L	2.8	0.30	23-Jan	st
4S	2.44	0.26	20.29	st
5L	3.03	0.64	39.22	m
5S	2.77	0.62	38.08	m
6L	3.03	0.93	48.19	m
6S	2.67	0.72	42.13	m
7L	2.42	0.72	41.91	m
7S	1.93	0.75	42.77	m
8L	2.46	0.28	21.73	st
8S	1.71	0.80	44.49	m
9_10L	2.38	0.26	20.80	st
9_10S	2.21	0.32	24.24	st

Chromosomal categories correspond to m = metacentric, sm = submetacentric, and st = subtelocentric chromosomes.

**Fig. 1:**
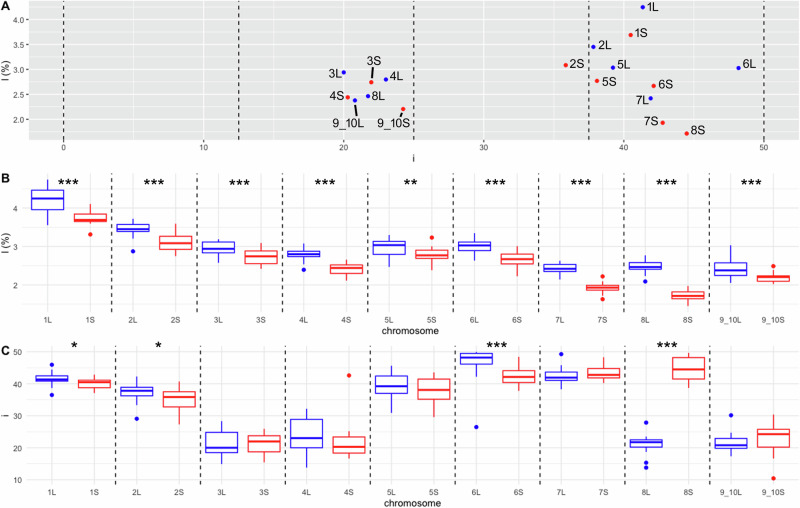
Analysis of measurements and statistics of the length of short and long arms of *X. pygmaeus* chromosomes. The L-subgenome is shown in blue and the S-subgenome in red. **A** Relationship between centromeric index (*i*), x-axis, and chromosome length (*l*), y-axis. Black dashed vertical lines delineate the intervals 0–12.5, 12.5–25, 25–37.5, and 37.5–50, corresponding to acrocentric, subtelocentric, submetacentric, and metacentric chromosomes, respectively. The plotted values of *i* and *l* are medians for each chromosome. Panel (**B**) shows intrachromosomal variation of *l* value (y axis) for the haploid complement of 18 *X. pygmaeus* chromosomes (x axis). **C** Intrachromosomal variability of *i* value (y axis) for the haploid complement of 18 *X. pygmaeus* chromosomes (x axis). **B**, **C** Black dashed vertical lines define pairs of homeologous chromosomes. Upper and lower whiskers show minimum and maximum values, respectively; boxes involve the lower (*Q*_1_) and upper (*Q*_3_) quartiles; horizontal lines inside the boxes indicate the median values (*Q*_2_); outliers are indicated by blue (for L) and red (for S) points above and below the whiskers. Significance codes for *l* and *i* values at the top of (**B**, **C**) define whether pairs of homeologous chromosomes are significantly different based on ANOVA and Tukey’s tests. Significantly different homeologs are depicted by significance codes ***, **, and * showing *p* values of *p* < 0.001, *p* < 0.01, and *p* < 0.05, respectively.

Subsequently, we plotted each chromosome on a graph based on *i* (x axis) and *l* (%, y axis) to determine whether pairs of homeologs had similar chromosomal morphology and whether they grouped closely together (Fig. 1A). The *l* and *i* values corresponding to each chromosome 1L–9_10S were plotted as well (Fig. 1B, C). A measure of statistical dispersion, the interquartile range (*Q*_1_–*Q*_3_), was used to evaluate the extent of morphological divergence of each pair of homeologous chromosomes. The highest divergence in *l* between homeologous chromosomes was found between chromosomes 8L and 8S. The *Q*_1_–*Q*_3_ of chromosomes 8L and 8S ranged from 2.41 to 2.58% and from 1.64% to 1.82%, respectively. The second highest divergence in *l* was measured within homeologous pairs 7L (2.35–2.53%) and 7S (1.87–1.99%) (Fig. 1B, Table S1). The largest difference in *i* between homeologous chromosomes was between chromosomes 8L and 8S, which differed by 20.2–22.5 and 41.5–48.2 within *Q*_1_–*Q*_3_, respectively. Each of homeologous chromosomes 8L and 8S was classified into different morphological category (st, and m; Table 2). The second greatest variation based on the *Q*_1_–*Q*_3_ *i* interval was between 6L (46.1–49.5) and 6S (40.4–44.1) (Fig. 1C, Table S2). One-way ANOVA and Tukey’s tests revealed statistically significant differences in *l* between all homeologous pairs and in *i* between chromosomes 1L and 1S, 2L and 2S, 6L and 6S, and 8L and 8S (Fig. 1B,C). This analysis confirmed the appropriateness of using the terms L as longer and S as shorter chromosomes of each homeologous pair.

### Mapping of 28S and 5S ribosomal genes

**Fig. 2:**
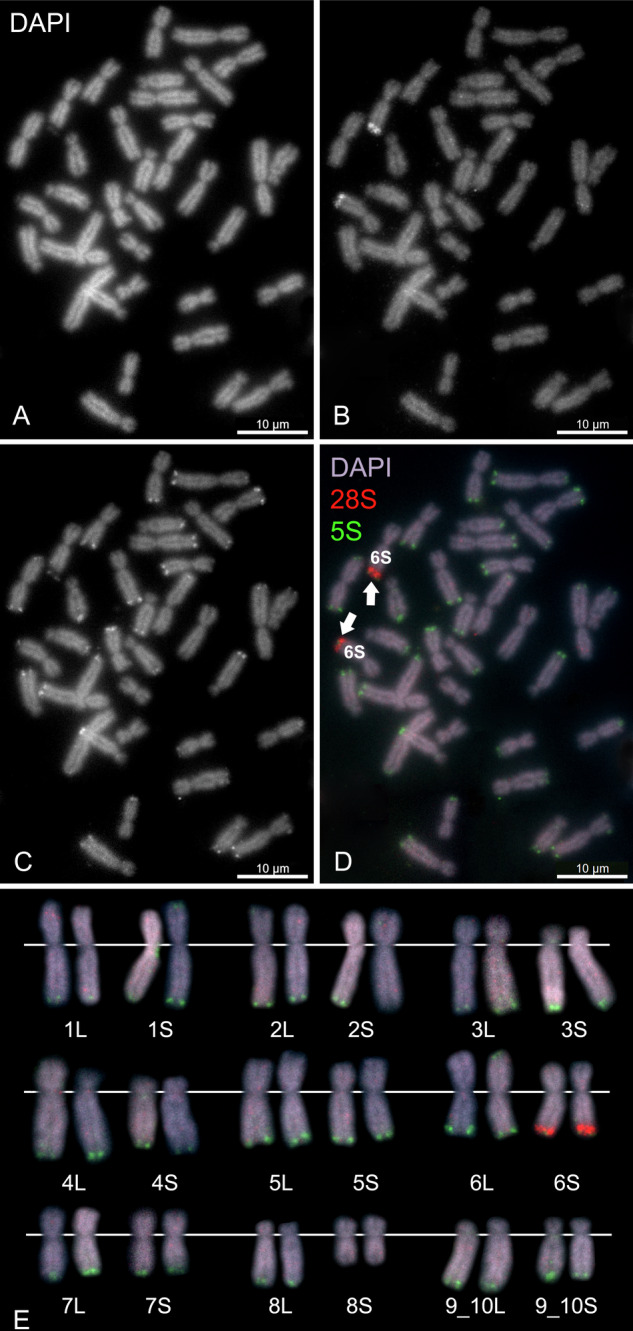
Mapping of ribosomal genes using FISH on metaphase spread in *X. pygmaeus*, sample XPYTaF1-1. **A** DAPI (black and white, B&W) counter-stained metaphase spread shows all 36 chromosomes. **B** FISH with the 28S rDNA probe (B&W), labeled with digoxigenin, reveals a signal on the long arm of chromosome 6S, indicating a nucleolar secondary constriction. **C** FISH with the 5S rDNA probe (B&W), labeled with biotin, highlights nearly all chromosomes in *X. pygmaeus* except chromosome 8S. **D** Merged metaphase (RGB) with DAPI (blue-purple) stain, 28S (red), and 5S (green) signals. Scale bars represent 10 μm. **E** Karyotype (RGB) arranged according to the *l* and *i* values. White lines indicate the position of the centromere.

In order to determine position and number of ribosomal genes in the genome we used FISH with 28S and 5S rDNA probes (Fig. 2). Using the 28S rDNA probe, we localized NORs. We expected to detect one pair of homologous NORs in allotetraploid *X. pygmaeus*, assuming that one pair of NORs would be inherited from an ancestor and one pair of NORs would be lost, as proposed in other tetraploids by Session et al. (2016), Knytl et al. (2017), Knytl et al. (2023). Indeed, we identified only one pair of NORs located on the telomere of the long arm of chromosome 6S, as depicted in Fig. 2B, D, E.

We localized the 5S rDNA loci in the telomeric regions of almost all chromosomes but one (8S, the shortest chromosome of the karyotype), as shown in Fig. 2C,D,E. Chromosome 2L bears the 5S rDNA signal on both arms. Chromosome 6S bears both 28S and 5S rDNAs—these two regions are spatially tightly adjacent to each other (visible on Fig. 2B, C). Chromosomes 4L and 5L have low-intensity signals, indicating a low copy number per 5S rDNA locus.

### Mapping of single-copy genes

Four to five plasmid clones from each amplified *X. pygmaeus* cDNA (*cept1*, *gyg2*, *fn1*, *ndufs1*, *sf3b1*, *nomo3*, *bmp7*, and *sox9*) were sequenced in order to distinguish homeologous copies. Sequences of all cDNA clones were aligned (for each gene separately) to compare their similarity to each other. Identity between clones was 99–100% except for *sox9* (93.35% identity between clones A and B). This finding confirmed that we amplified only one homeologous sequence for each gene except for the *sox9* gene, for which clones A and B are distinct homeologous copies. Each sequence was then compared to the *X. laevis* genome database (v.10.1) using BLASTn to determine which was the L and which was the S copy. Only one of two homeologous copies of *cept1* and *gyg2* is annotated in the *X. laevis* genome database. The *cept1* gene is annotated in the S-subgenome and the *gyg2* gene in the L-subgenome. Our *cept1* and *gyg2* cDNA sequences from *X. pygmaeus* had 98.7% and 90.64% identity with *cept1.S* and *gyg2.L* mRNAs from the *X. laevis* database, respectively. Because our amplified *gyg2* cDNA sequence had a lower similarity to *gyg2.L* from the *X. laevis* database than similarity between cDNA sequences within the same subgenome in these two species, it is possible that we amplified the *gyg2.S* and *cept1.S* copies. However in the following text, *cept1* and *gyg2* used as FISH probes are not specified as the L or S copies. Based on the similarity comparison between the *fn1*, *ndufs1*, *sf3b1*, *nomo3*, *bmp7*, and *sox9* sequences and the mRNA sequences of the same genes from the database, each of our sequences was assigned to either the L or S copy: *fn1*, *ndufs1*, *nomo3* and *bmp7* were assigned to the L copy, while *sf3b1* was assigned to the S copy. The similarity between our amplified *X. pygmaeus* sequences and the more similar homeologous *X. laevis* sequence was 95−98.5%. For the *sox9* gene both copies were amplified, clone A was identified as the S copy and clone B as the L copy. The L copy was selected for FISH-TSA (Table 1). Although we identified only one copy for most genes (our designed primers annealed to only one of two homeologous copies), this is likely due to the high divergence between the homeologous *X. pygmaeus* sequences, as well as the significant divergence between *X. pygmaeus* and *X. laevis*.

We previously identified the translocation of a massive heterochromatic block between chromosomes 9b and 2b in *X. mellotropicalis* (Knytl et al. 2017). The translocation involves the relocation of the *sf3b1* gene from chromosome 9b to 2b (or from chromosome 9 to 2 in a diploid ancestor) (Knytl et al. 2018). However, see Knytl et al. (2023) for the re-designation of the chromosome category from 2a to 2b. To test the hypothesis that the same translocation is also present in *X. pygmaeus*, we mapped five single-copy genes –*cept1*, *gyg2*, *fn1*, *ndufs1*, and *sf3b1*– that flank the translocated region in *X. mellotropicalis* (translocation-associated genes), as revealed by Zoo-FISH and FISH-TSA (Knytl et al. 2017, 2018). The *cept1* and *gyg2* genes are situated on the short (p) arm of chromosome 2 in *X. tropicalis*. In *X. laevis*, *cept1* and *gyg2* are situated on the long (q) and p arms of chromosome 2, respectively. Meanwhile the other three genes (*fn1*, *ndufs1* and *sf3b1*) are located on the q arm of chromosome 9 in *X. tropicalis* and the q arm of chromosome 9_10 in *X. laevis* (Knytl et al. 2018; Session et al. 2016; Uno et al. 2013).

We also tested whether the fusion between chromosomes 9 and 10 is also present in *X. pygmaeus*, as revealed in *X. laevis* (Session et al. 2016). To accomplish this, we selected three fusion-associated genes: *bmp7*, which is situated on the p arm of *X. tropicalis* chromosome 10, *nomo3* on the p arm of chromosome 9, and *sox9* on the q arm of chromosome 10 (Session et al. 2016; Uno et al. 2013). Three translocation-associated genes, *fn1*, *ndufs1*, *sf3b1*, were also used to detect the fusion between chromosomes 9 and 10. In addition, we hypothesized that the order of the localized genes would reveal the orientation of the ancestral chromosomes and fusion points.

**Fig. 3:**
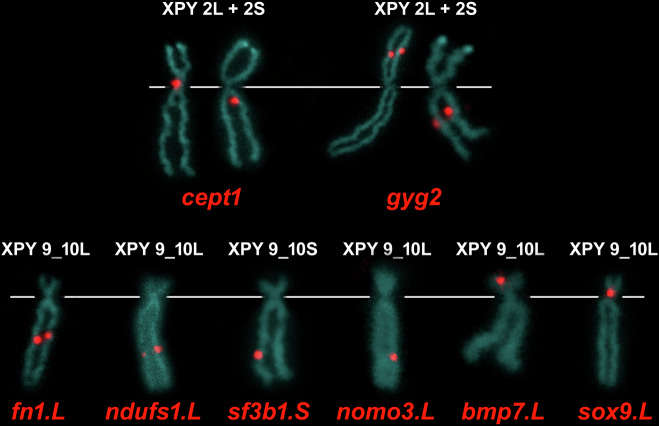
Mapping of single-copy genes by FISH-TSA with positive red signals on *X. pygmaeus* chromosomes (XPY), sample XPYTaF1-1. Each of the *cept1* and *gyg2* probes map to both *X. pygmaeus* chromosomes 2L and 2S. The *fn1.L*, *ndufs1.L*, *sf3b1.S* and *nomo3.L* loci are localized on the q arm of *X. pygmaeus* chromosomes 9_10L, 9_10L, 9_10S and 9_10L, respectively. The *bmp7.L* and *sox9.L* are mapped on the p arm of *X. pygmaeus* chromosomes 9_10L. White lines indicate the position of the centromere.

Translocation-associated genes in *X. pygmaeus*: the *fn1*, *ndufs1*, and *sf3b1* genes were localized on the q arm of chromosome 9_10 (Figs. 3, S3–S4) and the *cept1* and *gyg2* genes on chromosome 2 (Figs. 3, S1–S2). Thus, the translocation between chromosomes 9 and 2 did not happen in *X. pygmaeus* or its tetraploid ancestor. Interestingly, both *cept1* and *gyg2* mapped to the p arm of chromosome 2L and the q arm of chromosome 2S. Because *cept1* and *gyg2* mapped to non-homologous regions of chromosomes 2L and 2S (p and q arms), a pericentromeric inversion involving both *gyg2* and *cept1* genes occurred in the S-subgenome. Furthermore, the mapping analysis of the *gyg2* and *cept1* genes may improve the annotation of these two genes in the *X. laevis* genome assembly, in which one homeologous copy of each gene has not yet been annotated (*cept1.L* and *gyg2.S*). For more details, see Section Discussion, Evolution by genomic rearrangements.

Fusion-associated genes in *X. pygmaeus*: the *fn1*, *ndufs1*, *sf3b1* and *nomo3* genes were localized on the q arm of chromosome 9_10 (Figs. 3, S3–S4), while the *bmp7* and *sox9* genes were localized on the p arm of chromosome 9_10 (Figs. 3, S5). The order of the fusion-associated genes on *X. pygmaeus* chromosome 9_10 compared with the order of the same genes in *X. tropicalis* revealed that the ancestral chromosomes 9 and 10 fused through their q arms and that the centromere of chromosome 9 was lost and the centromere of chromosome 10 persisted (Fig. 3).

## Discussion

Although new amphibian genome assemblies have recently been generated (e.g., Bredeson et al. 2024; Dittrich et al. 2024; Holtz et al. 2023; Kuhl et al. 2024), the large size of amphibian genomes and the high cost of high-quality genome sequencing necessitate rapid cytogenetic alternatives for identifying rearrangements in chromosome structure. Using the available sequenced genomes of four African pipid frogs, *X. tropicalis* (Bredeson et al. 2024; Mitros et al. 2019), *X. laevis* (Session et al. 2016), *X. borealis* (Evans et al. 2022), and *Hymenochirus* sp. (Bredeson et al. 2024) as *H. boettgeri*, but see Gvoždík et al. (2024), it is possible to design primers for PCR and probes for FISH and test hypotheses as to whether any rearrangements occurred in species that have not yet had genomes sequenced. With the known evolutionary history of genus *Xenopus* (Evans et al. 2015), it is possible to estimate the dynamics of chromosome evolution, the ancestral chromosome structure, and the timing of rearrangements (Knytl et al. 2017, 2018, 2023). In this study, we used the genomes of *X. tropicalis* (subgenus *Silurana*) and *X. laevis* (subgenus *Xenopus*) to design FISH probes for a species from subgenus *Xenopus*, Pygmy clawed frog, *X. pygmaeus*, to explore orthologous synteny and add to the mosaic of knowledge about evolutionary dynamics of chromosomal rearrangements in *Xenopus*.

### Evolution by genomic rearrangements

The first step was to identify each *X. pygmaeus* chromosome based on measuring the p and q arms and calculating the *l* and *i* values to know which chromosome bears FISH signals. This detailed analysis of the *X. pygmaeus* karyotype has been performed for the first time, even though J. Tymowska published a karyotype of this species already in 1991.

For exploration of the timing of rearrangements, we included syntenic maps of *X. tropicalis*, *X. mellotropicalis* (both subgenus *Silurana*), *X. pygmaeus*, and *X. laevis* (both subgenus *Xenopus*) (this study; Knytl et al. 2018; Session et al. 2016; Uno et al. 2013). Genes for mapping were selected from the vicinity of already described interchromosomal rearrangements: the translocation (translocation-associated genes) discovered in subgenus *Silurana* (*X. mellotropicalis*; Knytl et al. 2017) and the fusion (fusion-associated genes) supposedly widespread in subgenus *Xenopus* (Session et al. 2016). Furthermore, intrachromosomal rearrangements – inversions were identified in the S-subgenome of *X. laevis* on chromosomes 2S, 3S, 4S, 5S, and 8S (Session et al. 2016).

**Fig. 4:**
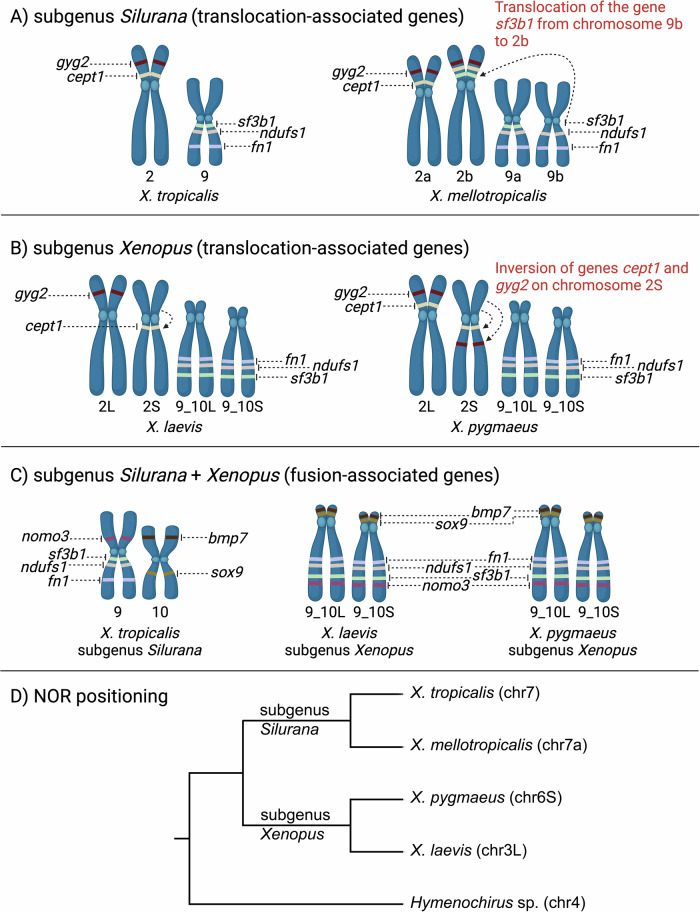
Locations of selected single-copy genes in four *Xenopus* species and phylogenetic relationships of these species with respect to NOR locations. The gene positions in *X. tropicalis* and *X. laevis* were taken from genome databases (Bredeson et al. 2024; Session et al. 2016). Gene localizations in *X. mellotropicalis* and *X. pygmaeus* were adopted from Knytl et al. (2018) and this study, respectively. Translocation-associated genes (*cept1*, *gyg2*, *fn1*, *ndufs1*, and *sf3b1*) are shown in subgenera *Silurana* (**A**) and *Xenopus* (**B**). **A** In *X. tropicalis* (*Silurana*), genes are considered to have ancestral positions. In *X. mellotropicalis*, the *sf3b1* gene was translocated from chromosome 9b to pericentromeric region of chromosome 2b (indicated by dashed arrow and described in red font). **B** In *X. laevis*, translocation-associated genes *cept1* and *gyg2* were annotated on one homeologous chromosome each, specifically on chromosome 2S and 2L, respectively. The remaining translocation-associated genes *fn1*, *ndufs1*, and *fn1* were annotated on both homeologous chromosomes 9_10L and 9_10S. In contrast, we mapped all translocation-associated genes on both homeologous chromosomes in *X. pygmaeus*. In addition, positions of the *cept1* and *gyg2* genes were inverted on chromosome 2S (indicated by dashed arrows and described in red font). **C** Fusion-associated genes (*fn1*, *ndufs1*, *sf3b1*, *nomo3*, *bmp7*, and *sox9*) depicted on *X. tropicalis* chromosomes 9 and 10, and *X. laevis* and *X. pygmaeus* chromosomes 9_10L and 9_10S. The order of the fusion-associated genes in these three species revealed that the fusion between chromosomes 9 and 10 is shared in *X. laevis* and *X. pygmaeus*. **D** A schematic phylogenetic tree showing variation in the NOR positioning. Two species from subgenus *Silurana* (*X. tropicalis*, *X. mellotropicalis*) and two species from subgenus *Xenopus* (*X. pygmaeus*, *X. laevis*) are shown for clear understanding of evolution by rearrangements depicted in A, B, and C. Chromosomes in parentheses associated with each species are NOR-carrying chromosomes (this study; Gvoždík et al. 2024; Knytl et al. 2017; Tymowska 1991). *Hymenochirus* sp. was used as an outgroup. **A**–**C** Created with BioRender.com, (**D**): created with Geneious Prime and modified in Adobe Photoshop.

Our mapping analysis of translocation-associated genes supported the hypothesis that the translocation between chromosomes 9 and 2 occurred solely in subgenus *Silurana* (Fig. 4A–B). However, it has yet to be investigated how widespread this translocation is among the different species within *Silurana*. The crucial species is *X. epitropicalis,* the sister species of *X. mellotropicalis*, and thus in what evolutionary period it likely occurred (Knytl et al. 2023).

Importantly, our mapping of *cept1* and *gyg2* probes revealed the inversion that occurred on chromosome 2S in *X. pygmaeus* (Fig. 4B). The large inversion was also identified on *X. laevis* chromosome 2S but not in *X. laevis* chromosome 2L or *X. tropicalis* chromosome 2 (Session et al. 2016). However, it was not clear whether the *cept1* and *gyg2* genes were involved in this inversion. Available genomic data showed the position of *cept1* exclusively on chromosome 2S and *gyg2* on chromosome 2L in *X. laevis*. The presence of only the S copy of the *cept1* in *X. laevis* is inconsistent with the hypothesis that the S-subgenome undergoes gene deletions and loses (Session et al. 2016), because the *cept1* would has been lost in the L-subgenome. Therefore, the missing *cept1.L* annotation may be due to a gap or sequencing artifact in the *X. laevis* genome assembly. To explore whether the *cept1* gene was involved in the inversion on *X. laevis* chromosome 2S, we compared the coordinates of the centromere position on chromosome 2S (54,347,978–55,190,352) (Smith et al. 2021) with the coordinates of the *cept1* gene (55,993,167–56,072,139) on the same chromosome (genome database). Consequently, the *cept1* gene is situated on the q arm of *X. laevis* chromosome 2S, as depicted in Fig. 4B, evidencing that *cept1* was indeed involved in the inversion. Because our FISH analysis revealed inversion in *X. pygmaeus* chromosome 2S that is homologous to the inverted region in *X. laevis*, this suggests that the inversion occurred in a common tetraploid ancestor of *X. pygmaeus* and *X. laevis* about 10 Mya (Evans et al. 2015, 2024; Fornaini et al. 2023, for evolutionary relationships see Fig. 4D), or even earlier in a common tetraploid ancestor of *X. pygmaeus*, *X. laevis*, and *X. borealis* or in a diploid ancestor of the S-subgenome (Knytl et al. 2024). However, whether this inversion is also present in other species of subgenus *Xenopus* remains to be investigated. Identifying its presence in additional species would help to more accurately date when the inversion occurred. Inconsistencies in the number of homeologous *cept1* and *gyg2* copies detected in *X. pygmaeus* by FISH and in *X. laevis* by inspection of the genome database may be caused by high divergence of these fragile regions, allowing enough time for the loss of one copy in *X. laevis*. Another explanation for this discordance is that both homeologous copies of the *cept1* and *gyg2* genes are present in the genomes of both *X. pygmaeus* and *X. laevis*.

The highest difference in the *l* and *i* values between homeologs was observed between *X. pygmaeus* chromosomes 8S and 8L. This finding highlighted chromosomes 8S and 8L as the most morphologically distinct homeologs in the karyotype. In *X. laevis*, the greatest divergence in *i* was also found between chromosomes 8L and 8S (Matsuda et al. 2015). Because both chromosomes 8S of *X. pygmaeus* and *X. laevis* include the pericentromeric inversion (this study; Knytl et al. 2024; Session et al. 2016; Uno et al. 2013), this indicates that *X. pygmaeus* and *X. laevis* share the same inversion, which likely caused the highest morphological difference between chromosomes 8L and 8S. Inversions in *Xenopus* appear to be significant drivers of divergent subgenome evolution and the re-diploidization process.

The location of fusion-associated genes in *X. pygmaeus* corresponds to the location of these genes in *X. laevis* (Session et al. 2016; Uno et al. 2013). The identical localization of these genes within both species confirms that the fusion of ancestral chromosomes 9 and 10 into chromosome 9_10 most likely occurred in a common diploid ancestor of subgenus *Xenopus*, before the divergence of the two subgenomes at least 30–35 Mya. The FISH mapping also revealed the fusion points and fate of centromeres in *X. pygmaeus* (Fig. 4C), which correspond to the same fusion discovered in *X. laevis* (Session et al. 2016) and *X. borealis* (Evans et al. 2022), and probably correspond to the fusion in all other species of subgenus *Xenopus* as all these species have chromosome numbers that are multiples of 18 (Tymowska 1991).

### Evolution of ribosomal genes and positions of NORs

The presence of 28S rDNA on one homologous pair of chromosomes in *X. pygmaeus* confirms the previously proposed hypothesis that one pair of homologous NORs has been lost in a tetraploid species/ancestor of genus *Xenopus*, since it is generally assumed that both diploid ancestors of allopolyploid species in both subgenera *Silurana* and *Xenopus* carried a pair of NORs. Hereafter in the text, one NOR equals a homologous pair of NORs, i.e. one NOR per each haploid complement. However, variation in NOR placement is inconsistent with the hypothesis that the *Silurana* a- and *Xenopus* L-subgenomes are more stable and the *Silurana* b- and *Xenopus* S-subgenomes tend to be more susceptible to rearrangements (Knytl et al. 2023; Session et al. 2016). *Xenopus laevis*, *X. gilli*, and *X. petersii* (then “X. species nova IX”; all three from the *X. laevis* species group) have NOR on the p arm of chromosome 3L (Roco et al. 2021; Session et al. 2016; Tymowska 1991; Tymowska and Kobel 1972). *Xenopus pygmaeus* and *X. parafraseri* (then “*X. fraseri*”, re-identified on the basis of the geographic origin “Foulassi, Cameroun”; both species from the *X. amieti* species group) have NOR on chromosome 6S (this study; Tymowska 1991). NOR in *X. clivii* (sister lineage to the *X. muelleri* species group) and *X. borealis* (*X. muelleri* species group) was detected on chromosome 4L (Schmid and Steinlein 2015; *X*. *borealis* genome database), and in *X. muelleri* (*X. muelleri* species group) on chromosome 5L (Schmid and Steinlein 2015; Tymowska 1991). Furthermore, the locations of NORs across subgenus *Silurana* differ from the locations of NORs in subgenus *Xenopus* (*X. tropicalis* has NOR on chromosome 7; *X. calcaratus*, *X. epitropicalis*, and *X. mellotropicalis* on chromosome 7a; Knytl et al. 2017, 2023; Tymowska 1991) and NORs evolved independently in each subgenus (Roco et al. 2021). NOR structures show highly dynamic character and tend to translocate from one chromosome to another across both subgenomes. Since all but one NOR position was identified in the L-subgenome, misidentification of chromosome 6S is possible. We used measurements of the *l* and *i* values, which are similar for chromosomes 5L, 5S, 6S and 7L in *X. pygmaeus* (Fig. 1B, C). An additional mapping marker co-localizing with NOR on the same chromosome is required to accurately identify the chromosome carrying NOR. Also, Tymowska (1991) identified NOR on *X. pygmaeus* chromosome 6S and therefore we are inclined to conclude that the NORs (not those ancestral ones that have been lost) have changed locations between chromosomes and subgenomes by a jump without prior deletion or duplication. The jumping mechanism supports the hypothesis that losses occurred in the *Xenopus* S-subgenome. We propose that the ancestral NOR was lost in the S-subgenome, and subsequently, the NOR jumped from the L-subgenome to the S-subgenome, as observed in contemporary *X. pygmaeus*. It is generally assumed that the jumping mechanism is a horizontal transmission of transposable elements caused by copy-and-paste or cut-and-paste transposition. These elements cause breaks on a chromosome and increase the probability of rearrangement (Castro et al. 2001; Chan et al. 2008; McClintock 1950; Rebollo et al. 2010). A similar mechanism that triggers the rearrangement of small nuclear DNA repeats in genus *Hymenochirus* was also suggested (Gvoždík et al. 2024). The jumping mechanism was also implicated in the context of horizontal translocation of sex-determining genes (e.g., Tennessen et al. 2018).

Taking the differences in NOR position and origin together, we are unable to clearly identify the ancestral position of NOR in subgenus *Xenopus*, although the ancestral position in subgenus *Silurana* is on chromosome 7 (Fig. 4D, Knytl et al. 2017, 2023; Tymowska 1991). Given the same NOR location in *X. clivii* and *X. borealis*, we can speculate that the ancestral NOR position in subgenus *Xenopus* is on chromosome 4L. The latter two species belong to the *X. muelleri* species group, and *X. clivii* is one of the most basal lineages of the subgenus. The sequence content of NORs is also worth investigating to find out if and what type of transposable elements surround the jumping NORs in genus *Xenopus*.

Another important characteristic is the copy number of repeats per locus. The copy number of repeats may be reduced in one subgenome but expanded in another, as proposed for *X. laevis* by Fornaini et al. (2023). In our focal species *X. pygmaeus*, we cannot count the number of 28S copies per locus, because its genome has not yet been sequenced. We consider that a low copy number per NOR locus can be undetectable by FISH mapping. The alternative that more NOR loci are present in the genome of *X. pygmaeus* than detected by our FISH method is less likely because only one NOR has also been identified in the genome of *X. laevis* (Session et al. 2016), as well as other cytogenetic studies have identified only one NOR in entire genus *Xenopus* (reviewed in Tymowska 1991). The pattern that only one NOR is present in the whole genome of most frogs, regardless of ploidy level (Gvoždík et al. 2024; Schmid et al. 2015; da Silva et al. 2024; Stöck et al. 2005; Tymowska 1991) indicates complete functional diploidization of these structures in allopolyploids. Exceptions among frogs include tetraploid *Odontophrynus americanus* (Odontophrynidae) and octoploid *Ceratophrys ornata* (Ceratophryidae), which show two NORs. The finding of two NORs indicates co-dominant expression of parental NORs and that their genomes have recently arisen from a single ancestral species via autopolyploidization (reviewed in Schmid et al. 2015). The results of the present study are consistent with the allotetraploid origin of *X. pygmaeus* and the loss of one ancestral NOR.

The 5S rRNA (along with the 5.8S, and 28S rRNAs) forms a large ribosomal subunit, the number of which varies among amphibians, but the location is generally telomeric (Knytl et al. 2017; Pardue 1974; Pardue et al. 1973). Using the FISH method, we found out that all but one *X*. *pygmaeus* chromosome carry 5S locus. There are only a few studies in which the 5S rDNA loci have been localized in genus *Xenopus*. *Xenopus laevis* has 5S rDNA signals on almost all chromosomes (Pardue et al. 1973) and, in contrast to *X. muelleri*, which has 5S rDNA locus on chromosome 6L (Schmid and Steinlein 2015). Species of subgenus *Silurana* have multiple 5S rDNA loci regardless of the diploid and tetraploid ploidy levels (Knytl et al. 2017, 2023). The number of 5S rDNA signals in *X. pygmaeus* is most similar to that of tetraploid *X. laevis* and diploid *X. tropicalis*. Given the high abundance of 5S rDNA loci detected across both *Xenopus* subgenera, we hypothesize that the presence of 5S rDNA loci on almost all chromosomes have been shared with their common ancestor for at least 45–50 Myr (Feng et al. 2017; Session et al. 2016), and the number of these loci has been duplicated by polyploidization events. However, in some species, such as *X. muelleri*, massive deletion of 5S rDNA has occurred (Schmid and Steinlein 2015).

### Sex chromosomes

All species of genus *Xenopus* have homomorphic sex chromosomes (Tymowska 1991), making cytogenetic investigation of sex chromosomes challenging. We could not identify the sex in our individuals, because we prepared chromosomes from young tadpoles. We analysed four individuals and found no differences in chromosome structure and morphology that would have indicated heteromorphy of sex chromosomes, thus confirming their homomorphy. *Xenopus pygmaeus* has chromosome 8L as a sex chromosome containing a sex-linked locus, which according to the coordinates of the *X. laevis* reference genome is located at 117–135 Mb, representing a telomeric region (Evans et al. 2024). None of the genes we mapped on *X. pygmaeus* chromosomes were situated on chromosome 8L except for the 5S locus. While the 5S locus on chromosome 8L mapped to the telomere, there is no indication that this 5S locus is associated with sex, given the presence of multiple 5S rDNA signals throughout the *X. pygmaeus* genome.

## Conclusion

Genus *Xenopus* is an exceptional model for evolutionary and genetics research due to its wide range of diverse genomic characteristics, such as one of the highest ploidy-level variations in animals (Tymowska 1991) and high variability in sex determination mechanisms (Evans et al. 2024; Furman et al. 2020; Song et al. 2021). Such variability can typically arise, for example, through various modes of asexual reproduction such as parthenogenesis (Dedukh et al. 2022), gynogenesis (Knytl et al. 2022), or hybridogenesis (Dedukh et al. 2020). However, none of these asexual modes of reproduction have been described in genus *Xenopus*. Therefore, there must be another mechanism that explains the exceptional genomic variation in these frogs. The most likely mechanism driving ploidy-level variation is hybridization between divergent ancestors, a hypothesis that has been repeatedly postulated and evidenced (e.g., Evans et al. 2005, 2015; Knytl et al. 2023; Session et al. 2016). However, the occurrence of natural hybrids is rare and documented only in closely related species (Kobel et al. 1981). In the present study, we found and highlighted that NORs in genus *Xenopus* are dynamic jumping structures and, interestingly, their number does not reflect ploidy level, as all species studied have a single NOR. Additionally, different tetraploid species have NORs on non-homologous chromosomes, demonstrating the jumping phenomenon. Nevertheless, some consistency in the NOR location is evident within subgenus *Silurana* and species groups of subgenus *Xenopus*, i.e. closely related species. The number and location of NORs in species with ploidy levels higher than tetraploid also need to be investigated. Interestingly, interchromosomal rearrangements, aside from the jumping NORs, are rare in genus *Xenopus*.

## Supplementary information

Supplementary Information

## Data Availability

The R scripts used for analysis of karyotype, including metaphase images, are available on the GitHub page (see Section Material and Methods, Analysis of karyotype). The DNA sequences have been deposited in GenBank, a publicly accessible database (see Table 1 for accession numbers). All other data supporting the findings of this study are available within the article and its Supplementary information.
