# Supplementary material for: FISH mapping in Xenopus pygmaeus refines understanding of genomic rearrangements and reveals jumping NORs in African clawed frogs
Source: Heredity (Edinb). 2025 Mar 1;134(3-4):209–20. doi: 10.1038/s41437-025-00749-x (PMC11977200; doi:10.1038/s41437-025-00749-x)
Supplement: Supplementary file 1 — Supplementary Information [file 41437_2025_749_MOESM1_ESM.docx]

**Supplementary information**

FISH mapping in *Xenopus* *pygmaeus* refines understanding of genomic rearrangements and reveals jumping NORs in African clawed frogs

Bergelová Barbora^1^, Gvoždík Václav^2,3^, Knytl Martin^1,4*^

^1^Department of Cell Biology, Faculty of Science, Charles University, Viničná 7, Prague 128 43, Czech Republic

^2^Institute of Vertebrate Biology of the Czech Academy of Sciences, Brno, Czech Republic

^3^National Museum of the Czech Republic, Department of Zoology, Prague, Czech Republic

^4^Department of Biology, McMaster University, 1280 Main Street West, Hamilton, Ontario, L8S 4K1, Canada

*Corresponding author. E-mail: [martin.knytl@natur.cuni.cz](mailto:martin.knytl@natur.cuni.cz)

**Table S1**. Minimum (*Q_1_*) and maximum (*Q_3_*) values, and interquartile range (IQR) of *l* values for each individual chromosome of *Xenopus pygmaeus*.

| Chromosome | Q1 | Q3 | IQR |
| --- | --- | --- | --- |
| 1L | 3.955738 | 4.463265 | 0.5075276 |
| 1S | 3.649400 | 3.842003 | 0.1926032 |
| 2L | 3.390865 | 3.571240 | 0.1803745 |
| 2S | 2.924742 | 3.264351 | 0.3396088 |
| 3L | 2.833916 | 3.115425 | 0.2815092 |
| 3S | 2.553159 | 2.883527 | 0.3303675 |
| 4L | 2.736651 | 2.875490 | 0.1388392 |
| 4S | 2.299303 | 2.520150 | 0.2208472 |
| 5L | 2.794647 | 3.132713 | 0.3380656 |
| 5S | 2.690328 | 2.903655 | 0.2133265 |
| 6L | 2.891630 | 3.114590 | 0.2229600 |
| 6S | 2.546554 | 2.801107 | 0.2545521 |
| 7L | 2.349426 | 2.532770 | 0.1833438 |
| 7S | 1.865308 | 1.990232 | 0.1249241 |
| 8L | 2.409116 | 2.580302 | 0.1711858 |
| 8S | 1.640418 | 1.818812 | 0.1783939 |
| 9_10L | 2.245852 | 2.573759 | 0.3279078 |
| 9_10S | 2.096166 | 2.235920 | 0.1397536 |

**Table S2**. Minimum (*Q_1_*) and maximum (*Q_3_*) values, and IQR of *i* values for each individual chromosome of *X. pygmaeus*.

| Chromosome | Q1 | Q3 | IQR |
| --- | --- | --- | --- |
| 1L | 40.82840 | 42.47758 | 1.649179 |
| 1S | 38.77390 | 41.12829 | 2.354390 |
| 2L | 36.23433 | 38.89834 | 2.664012 |
| 2S | 32.76803 | 37.51315 | 4.745116 |
| 3L | 18.49333 | 24.81203 | 6.318697 |
| 3S | 18.73691 | 23.75911 | 5.022201 |
| 4L | 20.00000 | 28.88610 | 8.886097 |
| 4S | 18.34563 | 23.38334 | 5.037712 |
| 5L | 36.99605 | 42.43417 | 5.438123 |
| 5S | 35.17231 | 41.46136 | 6.289053 |
| 6L | 46.14982 | 49.49495 | 3.345127 |
| 6S | 40.38147 | 44.08976 | 3.708286 |
| 7L | 41.07051 | 43.66062 | 2.590116 |
| 7S | 41.83428 | 44.75138 | 2.917097 |
| 8L | 20.20423 | 22.48062 | 2.276386 |
| 8S | 41.48809 | 48.18653 | 6.698435 |
| 9_10L | 19.84471 | 22.91469 | 3.069977 |
| 9_10S | 20.19870 | 25.77606 | 5.577352 |


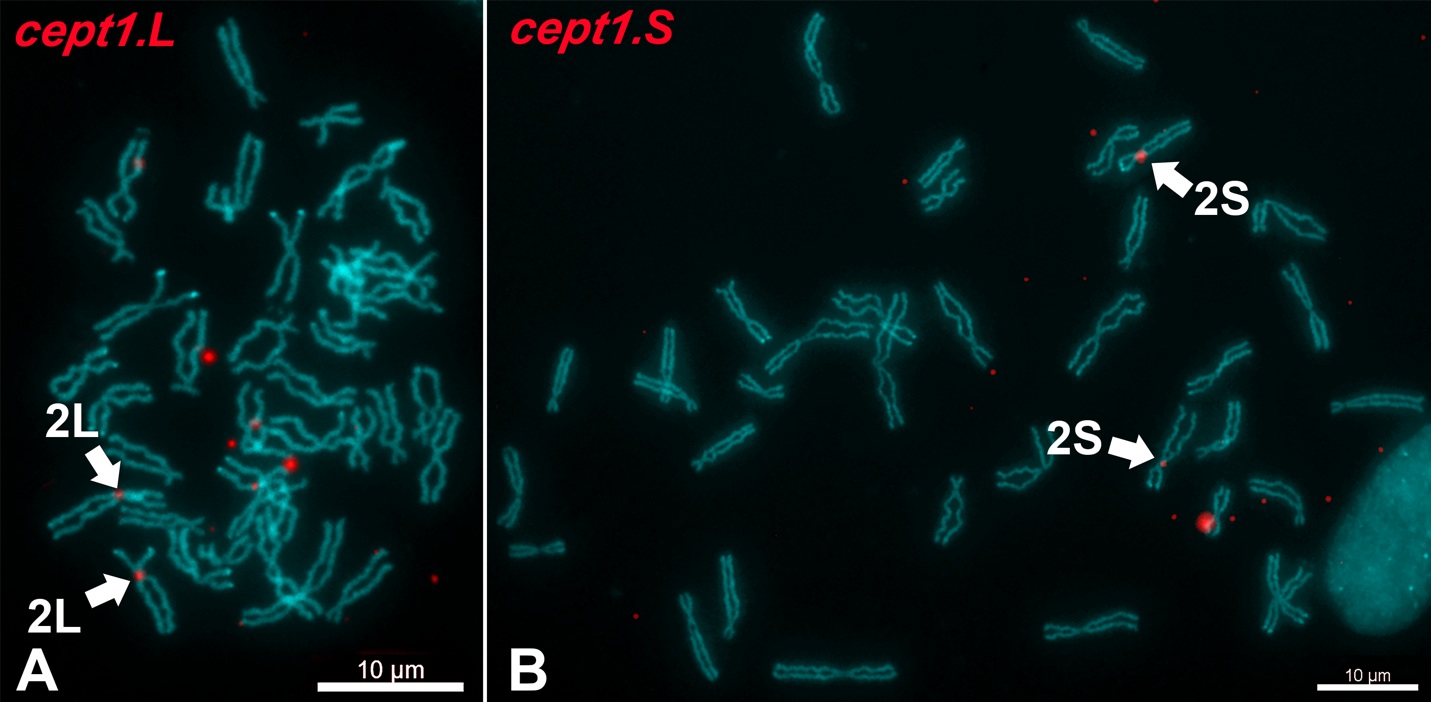


**Fig S1**. FISH-TSA with positive red signals on the *X. pygmaeus* metaphase spreads. (A) The *cept1.L* and (B) *cept1.S* genes were localized on the short (p) arm of *X. pygmaeus* chromosome 2L (XPY 2L) and long (q) arm of XPY 2S, respectively. Chromosomes were counterstained with DAPI (blue-green). Scale bars represent 10 µm.


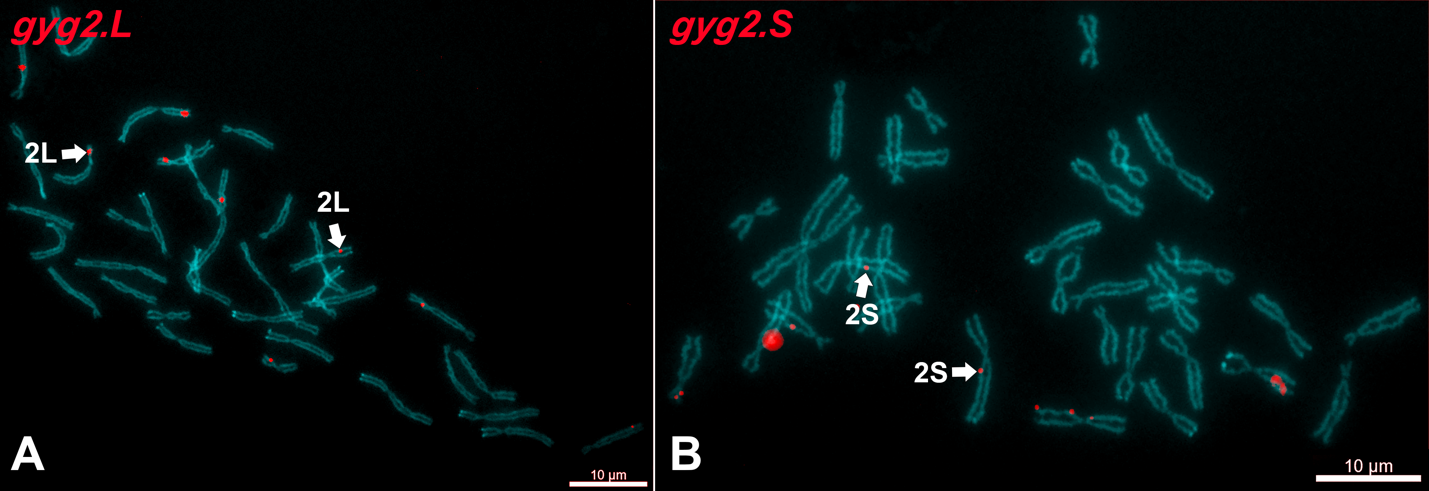


**Fig S2**. FISH-TSA with positive red signals on the *X. pygmaeus* metaphase spreads. (A) The *gyg2.L* and (B) *gyg2.S* genes were localized on the p arm of XPY 2L and q arm of XPY 2S, respectively. Chromosomes were counterstained with DAPI (blue-green). Scale bars represent 10 µm.


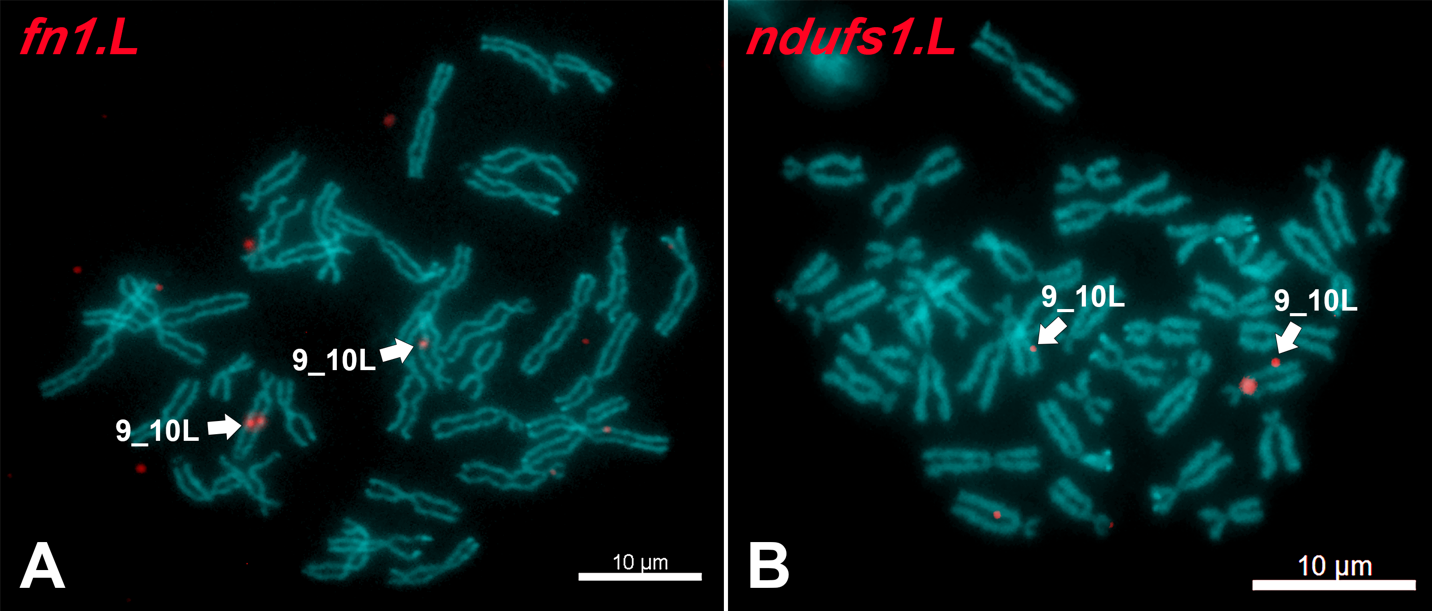


**Fig S3**. FISH-TSA with positive red signals on the *X. pygmaeus* metaphase spreads. (A) The *fn1.L* and (B) *ndufs1.L* genes were localized on the q arm of XPY 9_10L. Chromosomes were counterstained with DAPI (blue-green). Scale bars represent 10 µm.


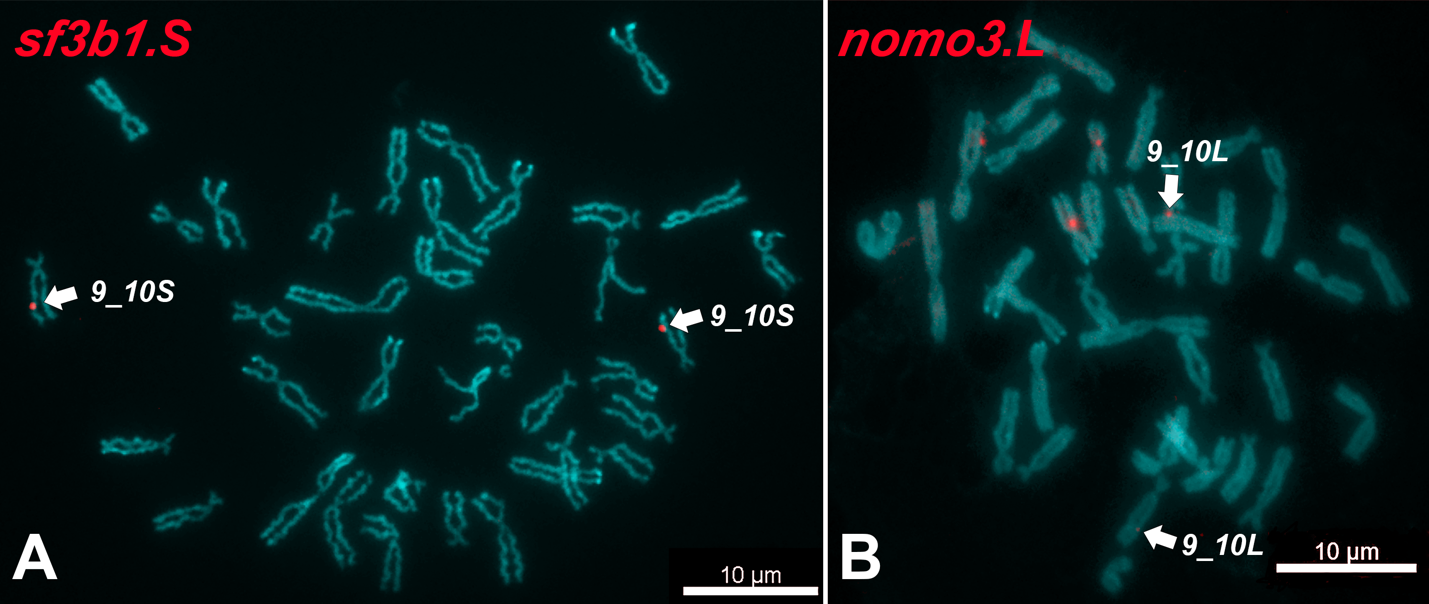


**Fig S4**. FISH-TSA with positive red signals on the *X. pygmaeus* metaphase spreads. (A) The *sf3b1.S* and (B) *nomo3.L* genes were localized on the q arm of XPY 9_10S and 9_10L, respectively. Chromosomes were counterstained with DAPI (blue-green). Scale bars represent 10 µm.


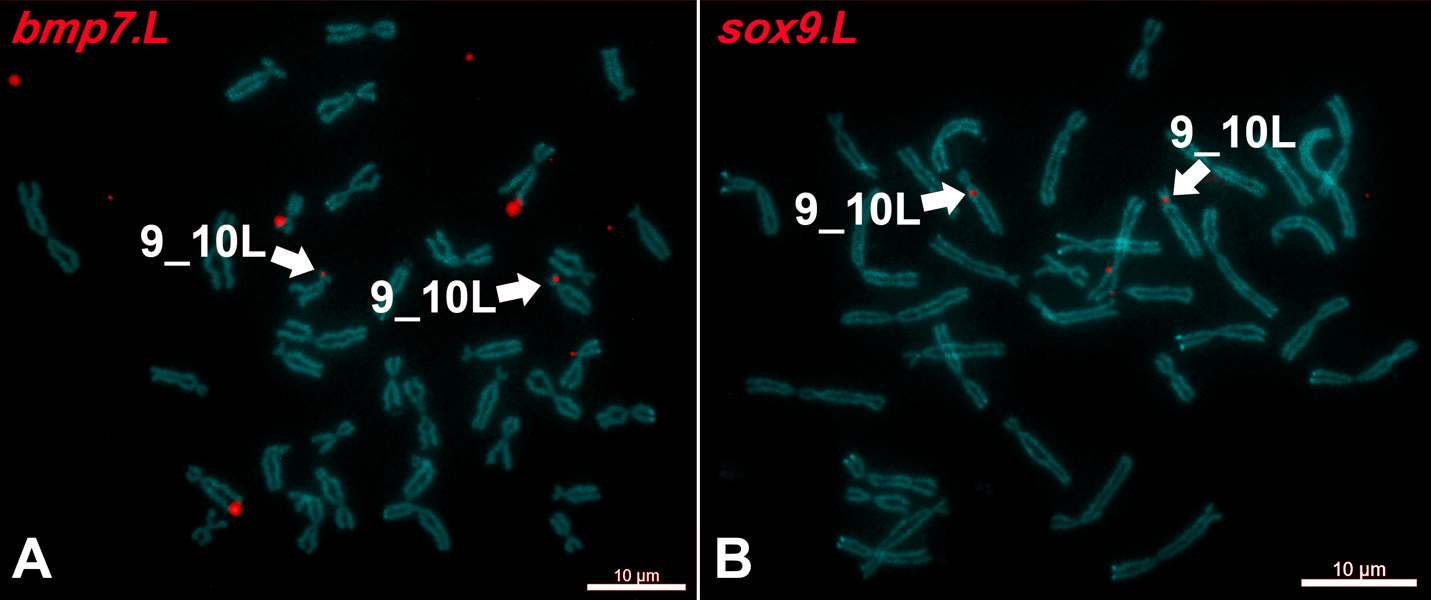


**Fig S5**. FISH-TSA with positive red signals on the *X. pygmaeus* metaphase spreads. (A) The *bmp7.L* and (B) *sox9.L* genes were localized on the p arm of XPY 9_10L. Chromosomes were counterstained with DAPI (blue-green). Scale bars represent 10 µm.
